# Tumor-intrinsic interferon signaling drives pancreatic cancer resistance to tumor mucin1-targeted CAR T cell therapy

**DOI:** 10.3389/fimmu.2025.1618415

**Published:** 2025-08-08

**Authors:** Ru Zhou, Rebecca Mayberry, Taina Firmin, Alexa Sanders, Cory Brouwer, John Maher, Pinku Mukherjee

**Affiliations:** ^1^ Department of Biological Sciences, University of North Carolina at Charlotte, Charlotte, NC, United States; ^2^ Department of Bioinformatics, University of North Carolina at Charlotte, Charlotte, NC, United States; ^3^ King’s College London, School of Cancer and Pharmaceutical Sciences, Guy’s Cancer Centre, London, United Kingdom

**Keywords:** CAR T cells, MUC1, PDA, resistance, interferon signaling

## Abstract

Pancreatic cancer (PC) remains one of the most challenging cancers and has the worst prognosis. Tumor-associated MUC1 (tMUC1) is overexpressed and aberrantly glycosylated in over 80% of human pancreatic ductal adenocarcinoma (PDA). Chimeric antigen receptor (CAR) engineered T cells are an emerging cancer immunotherapy strategy and recently, we successfully engineered tMUC1-specific human and mouse CAR T cells and demonstrated their effectiveness as monotherapy against PDA *in vitro* and *in vivo*. In this study, we observed varying sensitivity among human PDA cell lines in response to tMUC1-targeted CAR T cell cytolysis. Notably, highly resistant HPAFII cells released greater amounts of interferon (IFN)-regulated ICAM-1, CXCL10, and CXCL11 compared to the more sensitive MiaPaCa-2 cells following CAR T cell challenge. Blocking IFN signaling using Ruxolitinib, a JAK1/2 inhibitor (JAKi), significantly reduced the upregulation of ICAM-1 and CXCL10. Western blot analysis revealed that both type I and type II IFN signaling pathways were elevated in PDA cells upon CAR T cell treatment. JAKi effectively suppressed this signaling response, with a more pronounced impact on the type I IFN pathway. Importantly, both IFN blockade and transient knockdown of IFN receptors significantly enhanced the sensitivity of PDA cells to CAR T cell-mediated cytolysis *in vitro*. Further mechanistic study revealed that CAR T cells partially lose their cytolytic potential after engaging with PDA cells. Treatment with CAR T cells triggered the up-regulation of immune checkpoint PD-L1 expression on PDA cells via tumor cell’ own IFN signaling. Thus, blocking PD-L1 in HPAFII enhanced its response to CAR T cells. Similarly, neutralizing CXCL10 enhanced CAR T cell killing of HPAFII cells suggested CXCL10’s involvement in resistance to CAR T cell cytolysis. RNA-seq data indicated higher expression of multiple genes along the IFN signaling pathway which were associated with poor prognosis in PDA patients. Taken together, tumor intrinsic IFN signaling may drive immune evasion in PDA cells against tMUC1-targeted T cell-mediated immunotherapy. This identifies tumor IFN signaling as a potential therapeutic target to improve CAR T cell efficacy in PDA treatment.

## Introduction

Pancreatic cancer (PC) is currently the 3^rd^ leading cause of cancer death in the US and is projected to rise to 2^nd^ by 2040 (1). Among pancreatic cancers, pancreatic ductal adenocarcinoma (PDA) accounts for over 95% of cases and has a five-year relative survival rate of only 13% (2). This represents the poorest survival rate for any major epithelial malignancy, largely because most patients are diagnosed with advanced stage disease when symptoms appear (3). Given the overall low incidence rate of PC in the general population, screening for this disease has not been feasible. Approximately 75% of PDA patients present themselves with locally unresectable cancer. The mean expectation of life is less than six months and there are few long-term survivors because of the high rate of liver and peritoneal metastasis (4–7). For patients with resectable tumors, surgery offers the only single modality for potential cure. These patients have a two-year survival rate of 20-40% with surgery. Despite surgical resection, local recurrence or metastasis occurs in more than 50% of the patients. Adjuvant therapy including radiation and chemotherapy remains controversial in patients with resectable PDA. Very often cancer becomes resistant to such therapies.

MUC1 (CD227) is a transmembrane mucin glycoprotein that is normally expressed on all glandular epithelial cells of major organs including pancreas. As cells transform to a malignant phenotype, MUC1 is overexpressed and aberrantly glycosylated, known as tumor-associated form of MUC1 (tMUC1), and remains as an exciting target for immunotherapy (8, 9). The tMUC1 is expressed in over 80% of PDA cases (10–13), making it one of the most relevant cancer antigens for PDA. Our lab has developed a novel antibody, TAB004, that specifically targets tMUC1, distinguishing it from normal MUC1 (14–16).

Cancer immunotherapy has made revolutionary breakthrough in the treatment of hematological and solid malignancies, including immune checkpoint blockade (ICB) and chimeric antigen receptor (CAR) T cell therapies (17). By utilizing single-chain variable fragment (scFv) derived from TAB004, we successfully engineered tMUC1-targeted CAR T cells. TAB004 selectively targets tMUC1 while sparing normal MUC1, making it and derived CAR T cells safe for therapeutic use. tMUC1-specific CAR T cells have shown significant but variable efficacy against PDA *in vitro*, and the tumors were not completely eradicated *in vivo* (18). Despite the success of the emerging immunotherapies, resistance and tumor relapse remain major obstacles. Immune escape in solid tumors is frequently linked to mechanisms such as low tumor mutation burden (“immune-cold” tumors), MHC deficiency, lack of optimal tumor antigens, and immune checkpoint expression (19). Efforts to understand these resistance mechanisms have increasingly focused on the role of intrinsic tumor signaling.

Interferon (IFN) signaling has a complex role in cancer immunity and evasion. IFN effects vary depending on factors such as ligand concentration, signaling intensity and duration, tumor type, and prior treatments. Controversial data have been reported. The success of ICBs in several solid cancer types provides excitement (20). IFN-γ is believed to play an essential role in the success of ICB, and defects or deficiencies in the IFN-γ pathway are associated with resistance to anti-CTLA-4 therapy (21, 22). IFN-γ receptor (IFNGR) pathway is required for CAR T cell killing in solid but not liquid tumors. In glioblastoma cells, IFNGR1 was critical for sufficient CAR T cell binding to execute its productive cytotoxicity (23). However, IFN-γ also plays a role in tumor progression and metastasis. Low doses of IFN-γ promoted metastatic niche formation and enhanced tumor metastasis (24, 25). Sustained type I IFN signaling was observed in anti-PD-1-resistant tumors (26). Additionally, prolonged IFN-γ signaling promoted both PD-L1 dependent and independent resistance to ICB treatment in melanoma (27, 28). Furthermore, both type I and type II IFN pathways separately contributed to ICB-induced adaptive resistance (27). A recent genome-wide *in vivo* CRISPR screen identified IFN-induced upregulation of classical and non-classical MHC class I inhibitory checkpoints as tumor-intrinsic immune evasion mechanisms (29). Loss of tumor-intrinsic IFN-γ signaling was shown to sensitize tumors to immune responses, with strong IFN signatures correlating with poor ICB outcomes in renal cell carcinoma and melanoma. Taken altogether, the role of cancer intrinsic IFN signaling in antitumor immunity and tumor escape remains to be further elucidated.

Disease resistance and refractory status are two major hurdles in pancreatic cancer treatment (30). Here, our study demonstrated that tumor IFN signaling contributed to PDA resistance against tMUC1-targeted CAR T cell therapy. We found that pretreating PDA cells with Janus kinase (JAK1/JAK2) inhibitor or knocking down IFN receptors significantly enhanced PDA sensitivity to CAR T cell cytolysis *in vitro*. RNA-seq data analysis using TCGA database suggested a correlation of higher gene expressions in tumor IFN pathway with poor survival in patients with PDA. Therefore, disrupting tumor IFN signaling may break PDA resistance and improve the efficacy of CAR T cell therapy in PDA patients.

## Materials and methods

### Culture media

The DMEM or MEM were supplemented with 10% fetal bovine serum (FBS; R&D Systems, Minneapolis, MN), 1% glutamax, 1% non-essential amino acids, and 1% penicillin/streptomycin as complete medium. The RPMI1640 was supplemented with 10% heat inactivated FBS, 1% glutamax, 10mM HEPES, and 1% penicillin/streptomycin. All the medium and supplements were obtained from Thermo Fisher Scientific (Waltham, MA) if not separately mentioned.

### Cell culture

Selected human pancreatic cancer cell lines (HPAC, Capan2, PANC-1 MiaPaCa-2, CFPAC, HPAFII) were obtained from American Type Culture Collection (ATCC, Manassas, VA 20110, USA) and were cultured as instructed. The retroviral vector packaging line GP2–293 cell line was cultured in complete DMEM.

### Tumor MUC1 detection by flow cytometry

The tMUC1 expression on human PDA cell lines was assessed by TAB004 staining (1μg/sample; provided by OncoTab Inc., Charlotte, NC) conjugated with HiLyte Fluor 647 (Dojindo Molecular Technologies, Inc., Rockville, MD), named as TAB004-Fluor 647. Dead cells were excluded by 7-AAD staining (BD Biosciences). Data was acquired on BD LSRFortessa flow cytometer (BD Biosciences), and analyzed with FlowJo software (version 10.8.1, Tree Star Inc).

### Construction of chimeric antigen receptor and retroviral supernatant production

The second-generation tMUC1-specific human CAR construct was synthesized by Dr. John Maher group and the schematic of MUC1 CAR was detailed in (31). The monoclonal antibody TAB004 was developed against tumor-associated form of human MUC1 (14). A Myc epitope-tagged framework was engineered to enable the monitoring of tMUC1-specific CAR expression.

To produce the tMUC1 CAR-encoding retroviral supernatants, GP2–293 cells were transfected with the SFG-retroviral CAR plasmid and VSV-G plasmid via Effectene following the manufacturer’s instruction (Qiagen, Germantown, MD). Virus supernatants were collected 48h and 72h after transfection and stored at -80°C.

### CAR T cell transduction

Human PBMCs from various healthy donors (purchased from STEMCELL Technologies, Cambridge, MA) were activated with anti-CD3 (5μg/ml, plate-bound) andanti-CD28 (2μg/ml, soluble) antibodies (ThermoFisher Scientific, Waltham, MA). On day 3, activated T cells were transduced with the CAR retroviral supernatants and spinoculated at 32 °C for 1 hour at 2,000g in 6-well non-tissue culture plates coated with Retronectin (Takara, Mountain View, CA). Transduced T cells were maintained in recombinant human IL-2 (10ng/ml, PeproTech, Rocky Hill, NJ). On the next day, the culture supernatants were replaced with fresh complete RPMI1640 plus IL-2. The transduced T cells were passaged, and fresh media was supplied when appropriate. The CAR transduction efficiency was monitored by flow cytometry analysis. Activated but non-retroviral transduced cells were included as Mock T cell control. CAR T cells were used for functional assays on day 7–15 post transduction.

### Expression of CAR detected by flow cytometry

The human tMUC1-CAR expression was assessed via Myc-tag-AF488 staining (Cell Signaling Technologies, Danvers, MA). T cell phenotypes were determined by staining for CD4-PE/Cy7 and CD8-eF450 (BioLegend, San Diego, CA). Dead cells were excluded by 7-AAD staining (BD Biosciences). Data was acquired on BD LSRFortessa flow cytometer, and analyzed with FlowJo software

### CAR T cell cytotoxicity

The antigen-specific tumor cell lysis by tMUC1-CAR T cells was determined using MTT assay. Briefly, a panel of PDA cell lines (10,000 cells/well) were plated in 96-well plates overnight. The next day, culture media from the plate was aspirated and CAR T cells or Mock T cells were added at the E:T ratio of 5:1. After co-culture for 24hr, T cells were washed with phosphate buffered saline (PBS) and removed and replaced with fresh media containing MTT (500μg/ml, Sigma) for 3hr. After the uptake of MTT, supernatants from wells were discarded and 150μl of DMSO was added to wells to dissolve the formazan crystals. The OD value was read at 540nm. When we used the mock T cell lysis data for calculating % lysis, the calculation formula was: [(OD of co-culture with Mock T cells – OD of co-culture with CAR T cells)/OD of co-culture with Mock T cells] ×100. When we used the media control data for calculating % lysis, the calculation formula was: [(OD of culture with media – OD of co-culture with CAR T or Mock T cells)/OD of culture with media] ×100. The exact applied formula was described in the relevant Figure Legends.

The CAR T cell lysis against PDA with Ruxolitinib (JAKi; R&D Systems; Minneapolis, MN) pretreatment or with IFN receptor knockdown were separately described in Figure Legends.

### Cytokine detection

The antigen-specific cytokine release was performed by co-culturing tMUC1-CAR T cells with PDA cells at E:T ratio of 5:1. After 24hr of co-culture, supernatants were collected. The panel of cytokines was detected using Proteome Profiler Human Cytokine Array Kit, Panel A (R&D Systems; Minneapolis, MN) as instructed. The array data were quantitated by ImageJ software. The cytokine release under other treatment conditions was separately described in Figure Legends. The individual cytokine concentration was determined using Duoset Human Cytokine Detection Kit in accordance with the manufacturer’s instructions (R&D Systems).

### Western blot

Western blot (WB) was performed as previously described (16). Briefly, PDA cells were treated as detailed in Figure Legends. Cells were pelleted and suspended in the lysis buffer (20 mM Tris–HCl, 150 mM NaCl, 1 mM EDTA, 1% Triton X-100), supplemented with Halt protease and phosphatase inhibitor cocktail (Thermo Fisher Scientific) for 15min at 4°C. The cell lysates were centrifuged at 13,000 rpm for 5min at 4°C. Protein supernatants were collected and quantified using BCA assay (Thermo Fisher Scientific). The supernatants were boiled in the SDS sample buffer [50 mM Tris–HCl (pH 6.8), 2% SDS, 10% glycerol, 1.2% 2-mercaptoethanol and 0.02% bromphenol blue] for 5 min at 96°C. Equal amounts of proteins (10µg to 30µg per lane) were electrophoresed in 7%, 10%, or 12% polyacrylamide gel and then transferred to PVDF membranes (Bio-Rad, Hercules, CA, USA). The membranes were treated with 10% non-fat milk for 1hr to block non-specific binding, then rinsed and incubated with various antibodies. All primary antibodies purchased from Cell Signaling Technologies were diluted at 1:1000 (Cat# 35114T, Cat # 44902T, Cat# 53883, Cat# 94994S, Cat#9139S), except that the anti-IFNalpha/beta receptor 1 antibody was diluted at 1:2000 (Cat# ab245367; Abcam, Waltham, MA). The membranes were then treated with 1:1000 dilution of horseradish peroxidase-conjugated anti-rabbit or anti-mouse IgG (Cell Signaling Technologies) for 1hr. Immune complexes were detected with a chemiluminescence substrate (Cat# 34076; Thermo Fisher Scientific) and exposed using ChemiDoc MP Imaging System (Bio-Rad). The density of signal was quantified by ImageJ and presented along with the blots.

### IFN receptor knockdown by siRNA transfection

HPAC, Capan2, MiaPaCa-2 and HPAFII cells were plated at 100, 000 cells/well in 24-well plates and grown to 50-70% confluence. Cells were transfected with human IFN receptor-specific siRNA (Silence select IFNAR1/IFNAR2/IFNGR2) or control siRNA (Silencer Select Negative Control No. 2 siRNA) (Thermo Fisher Scientific) in Lipofectamine 3000 transfection reagent (Thermo Fisher Scientific), in accordance with the manufacturer’s instructions. The knockdown of IFN receptors was verified 48hr and 72hr after siRNA transfection.

### Cytotoxicity of CAR T cells post CAR T-PDA co-culture

On day 1, MiaPaCa-2 and HPAFII cells were plated overnight for attachment. On day 2, CAR T cells or Mock T cells were cultured with these two PDA cells at E:T ratio of 5:1 or cultured with media alone for overnight. On the same day, fresh MiaPaCa-2 and HPAFII cells were plated overnight for attachment. On day 3, the live CAR T cells and Mock T cells were isolated from the day-2 cultures using Lympholyte-M Cell Separation Media (Cedarlane, Burlington, NC). Thus, three pairs of live T cells were prepared: Mock T cells and CAR T cells from 1) culture with media alone 2); from culture with MiaPaCa-2 3); from culture with HPAFII. These three pairs of T cells were added to the fresh MiaPaCa-2 or HPAFII plates (prepared on day-2) at E:T ratio of 2:1 or 5:1 for 24hr. On day 4, CAR T cell lysis against MiaPaCa-2 or HPAFII was determined using MTT assay. The percentage of lysis was calculated using the formula: [(OD of co-culture with Mock T cells– OD of co-culture with CAR T cells)/OD of co-culture with Mock T cells] ×100. And the lysis was calculated using the Mock T cells and CAR T cells from the same pair.

### Expression of activation marker and immune checkpoints (ICs) on CAR T cell surface

MiaPaCa-2 and HPAFII cells were plated overnight for attachment. On the following day, CAR T cells were added to PDA culture at E:T ratio of 5:1 or cultured with media alone for overnight. Then CAR T cells from culture were collected and stained for Myc-tag-AF488, CD45-APC/Cy7, CD4-PE/Cy7, CD8-eF450, CD25-PE, PD-1-PE, TIM-3-PE, LAG-3-PE, TIGIT-APC, and 7-AAD. All the antibodies used here for flow cytometry were purchased from BioLegend, except for Myc-tag-AF488 and 7-AAD.

### Expression of tMUC1 and PD-L1 post CAR T cell challenge

MiaPaCa-2 and HPAFII cells were plated overnight for attachment. On the following day, PDA cells were treated with CAR T cells at E:T ratio of 5:1 or cultured with media alone for overnight. After gently rinsing off suspension cells with PBS in co-culture, adherent MiaPaCa-2 or HPAFII cells were collected and stained with CD45-APC/Cy7, TAB004-Fluor 647, PD-L1-PE (BioLegend), and 7-AAD. CD45 staining was used to exclude non-tumor cells from analysis.

### Inhibition of PD-L1 with JAKi

MiaPaCa-2 and HPAFII cells were plated overnight for attachment. On the next day, PDA cells were treated with JAKi at 25μM for overnight. Then JAKi was removed, and CAR T cells were added in the absence of JAKi. CAR T cells were added at E:T ratio of 5:1, calculated based on the initial number of PDA cells plated. After 24hr treatment with CAR T cells, live adherent PDA cells were collected and stained with CD45-APC/Cy7, PD-L1-PE, and 7-AAD.

### Binding efficiency of PD-L1 blocking antibody

HPAFII cells were plated overnight and followed by CAR T cell treatment at E:T ratio of 5:1 for another overnight. After gently rinsing off suspension cells with PBS, adherent HPAFII cells were collected, resuspended in flow cytometry buffer, and aliquoted into 100μl cell suspension per staining tube. PD-L1 blocking antibody (Cat# 329716; BioLegend) were added at 10μg/ml or 100μg/ml in the respective tubes and incubated for 30min at 4 °C. The control tube was added equal volume of PBS. After 30min incubation, cells were washed with flow cytometry buffer to remove unbound PD-L1 blocking antibody. Then cells were resuspended and stained with CD45-APC/Cy7, PD-L1-PE, and 7-AAD.

### Effect of blocking or neutralizing antibodies on CAR T cell cytotoxicity against PDA

MiaPaCa-2 and HPAFII cells were plated overnight for attachment. On the following day, PDA cells were pre-incubated with PD-L1 blocking antibody (10μg/ml) or its isotype control antibody (10μg/ml; Cat# 400348; BioLegend), anti-ICAM-1 neutralizing antibody (5μg/ml; Cat# AF720; R&D Systems), anti-CXCL10 neutralizing antibody (5μg/ml; Cat# MAB266-100; R&D Systems), or fresh media alone for 2hr. Then without removing antibodies, CAR T cells were added at E:T ratio of 5:1 or cultured with media alone for 24hr. MiaPaCa-2 or HPAFII cell lysis was determined using MTT assay. The percentage of lysis was calculated using the formula: [(OD of co-culture with Mock T cells– OD of co-culture with CAR T cells)/OD of co-culture with Mock T cells] ×100.

### Gene expression-survival analysis

RNA-seq gene count data in the TCGA (The Cancer Genome Atlas) pancreatic adenocarcinoma project were downloaded from the GDC (Genomics Data Commons Portal) (32, 33). A total of 183 pancreatic tumor samples were included in the analysis. Gene expression was calculated as TPM (transcripts per million) from STAR alignment counts (34). For each gene, samples are considered to have low expression if the value was below the median gene expression and high expression if above. Survival curves were generated from the resulting dataset using the survival package in R and significant differences between the two groups were also calculated using the default parameters according to the documentation (35, 36).

### Statistical analysis

Data were analyzed using Prism (version 10; GraphPad Software) and results were presented as mean ± SD or mean ± SEM. For all the cytotoxicity assay, individual values were also included in figures and shown using symbols. Data were representative of two or more independent experiments. The calculation of significance was described in Figure Legends.

## Results

### Different sensitivity of PDA cells to tumor MUC1-targeted CAR T cell cytolysis *in vitro*

The expression of tMUC1-CAR on activated human T cells was shown in **Supplementary Figure 1**. On day 7 post-retrovirus transduction, 32.5% of CD4^+^T cells and 16.3% of CD8^+^T cells were Myc-tag-positive. The whole transduced T cells were used for this study.

**Figure 1:**
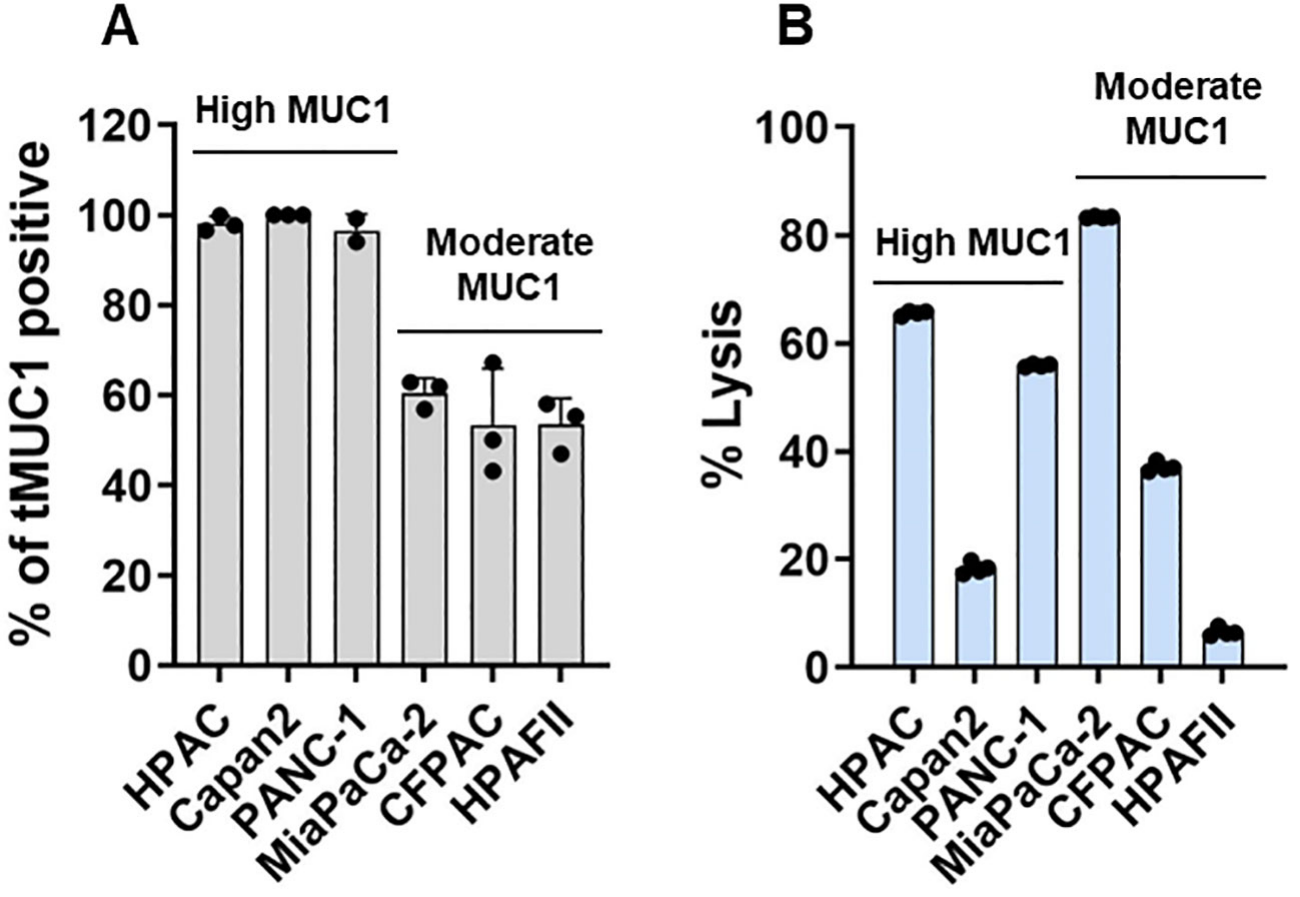
Human tMUC1-CAR T cells target and lyse PDA cells *in vitro.* **(A)** Percentage of PDA cells expressing human tMUC1, as determined by TAB004-Fluor 647 staining and flow cytometry analysis. Dead cells were excluded by 7-AAD staining. Data are presented as mean ± SEM from 3 separate experiments. Individual values are included. **(B)** Percentage of PDA cell lysis by tMUC1-CAR T cells. CAR T cells and PDA cell lines were co-cultured at E:T ratio of 5:1 for 24hr. Tumor cell lysis was determined using MTT assay. The percentage of lysis was calculated using the formula: [(OD of co-culture with Mock T cells– OD of co-culture with CAR T cells)/OD of co-culture with Mock T cells] ×100. Data are presented as the mean ± SD from quadruplicate.

First, human PDA cell lines were analyzed for the levels of tMUC1 on their cell surface by flow cytometry. Data were presented as percentage of cells that expressed tMUC1 (**Figure 1A**). PDA cells were categorized into two groups based on their tMUC1 expression levels: tMUC1-high and tMUC1-moderate expressing cells. Next, the efficacy of CAR T cell cytolysis against PDAs was shown as **Figure 1B**. PDA cells were co-cultured with CAR T cells at a E:T ratio of 5:1 for 24hr. The tumor lysis data of CAR T cells was normalized to the corresponding mock T cell control for each PDA cell line. CAR T cells were able to kill tMUC1-positive PDA cell lines (**Figure 1B**). However, the sensitivity of PDAs to CAR T cell-mediated killing varied and was not strictly correlated with their tMUC1 levels. Among the tMUC1 high-expressing PDAs, Capan2 exhibited significantly lower sensitivity to CAR T cell killing compared to HPAC. Similarly, within the tMUC1 moderate-expressing PDAs, HPAFII displayed strong resistance to CAR T cell killing compared to MiaPaCa-2. Overall, PDA cell lines demonstrated variable resistance to CAR T cell cytolysis.

### IFN-related immune factors indicate a potential link between IFN signaling and PDA resistance

**Figure 2:**
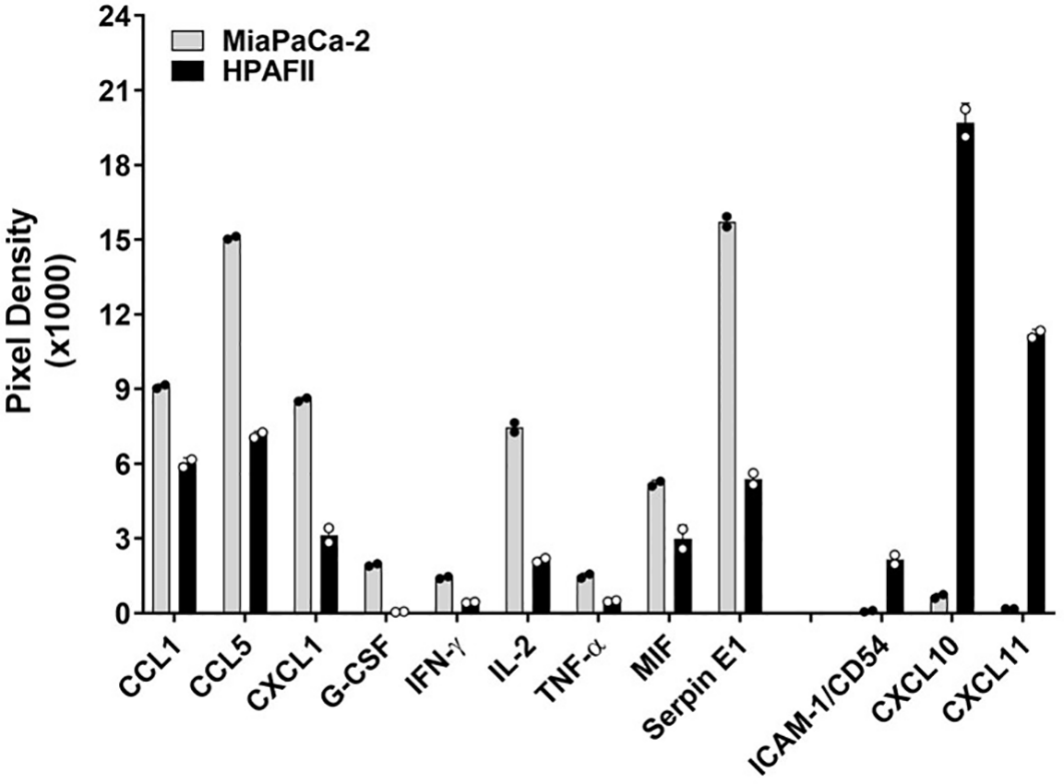
Differential cytokine release. MiaPaCa-2 and HPAFII cells were co-cultured with human tMUC1-CAR T cells at E:T ratio of 5:1 for 24hr. The co-culture supernatants were analyzed by human cytokine array. Data are presented as mean ± SD of replicate. Replicate values are shown as well.

To investigate the mechanisms underlying the strong resistance observed in HPAFII cells, we co-cultured the highly sensitive MiaPaCa-2 cells and the highly resistant HPAFII cells with CAR T cells at a E:T ratio of 5:1 for 24hr. Following the co-culture, supernatants were analyzed using a human cytokine array to identify differentially released cytokines and chemokines (**Figure 2**), in which the focus was given to what was higher in the co-culture with HPAFII cells. We observed significantly higher levels of the adhesion molecule ICAM-1 and the chemokines CXCL10 and CXCL11 in the HPAFII-CAR T cell co-culture compared to the MiaPaCa-2-CAR T cell co-culture. ICAM-1, CXCL10, and CXCL11 are predominantly induced by IFN-γ (37–39), though they can also be triggered by type I IFNs (40). The elevated release of these IFN-related factors in the HPAFII-CAR T cell co-culture suggested a potential correlation between higher tumor-associated IFN activation and stronger PDA resistance to CAR T cell killing.

### Blocking IFN signaling in PDA reduces its IFN-associated cytokine release

**Figure 3:**
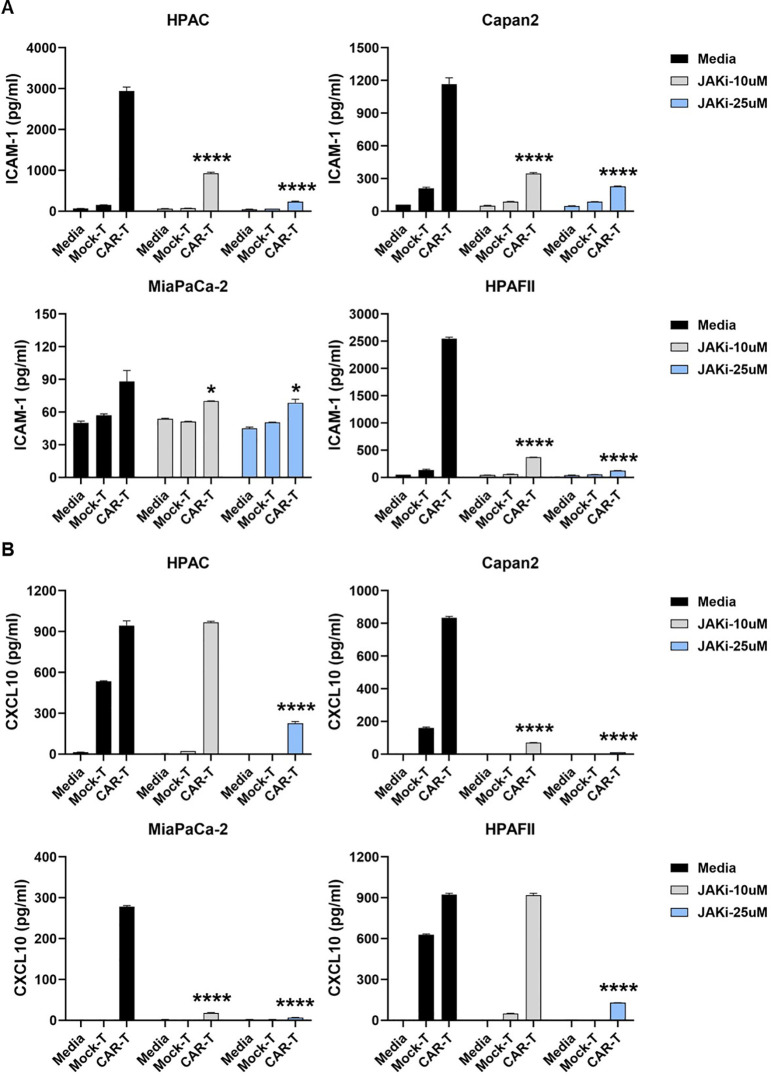
Suppressing IFN signaling in PDA reduces ICAM-1 and CXCL10 levels. PDA cell lines were plated overnight for attachment. On the following day, PDA cells were treated with JAKi at 10μM and 25μM overnight. After JAKi treatment, it was removed, and tMUC1-CAR T cells or Mock T cells were added at E:T ratio of 5:1 in the absence of JAKi. The E:T ratio was calculated based on the initial number of PDA cells plated. After 24hr, co-culture supernatants were collected and assayed for cytokine levels of **(A)** ICAM-1 and **(B)** CXCL10 by ELISA. Data are presented as the mean ± SD of triplicate. Baseline levels of ICAM-1 and CXCL10 were low (<50pg/ml and <10pg/ml, respectively) in either CAR T cell-only or in PDA-only cultures, which were detected by ELISA. The statistical comparison was conducted between CAR T cell treatment in JAKi-pretreated PDA and CAR T cell treatment in PDA without JAKi pretreatment. *p<0.05, ****p<0.0001 (unpaired t test with Welch’s correction).

Ruxolitinib, a selective JAK1/2 kinase inhibitor known to suppress JAK-STAT signaling (41), was used to assess the role of tumor-intrinsic IFN signaling in the elevated release of ICAM-1 and CXCL10. The cytotoxic effect of Ruxolitinib on PDA cells was first determined (**Supplementary Figure 2**). To investigate the impact of IFN signaling within PDA cells, we pre-treated PDA cells with or without Ruxolitinib (briefly as JAKi, at 10μM or 25μM) overnight. Following treatment, JAKi was removed, and CAR T cells were added for co-culture with PDA cells for 24hr. Analysis of the co-culture supernatants revealed a significant reduction in ICAM-1 and CXCL10 levels when PDA IFN signaling was pre-disrupted, with the exception of ICAM-1 in the highly sensitive MiaPaCa-2 cell line (**Figures 3A, B**).

**Figure 4:**
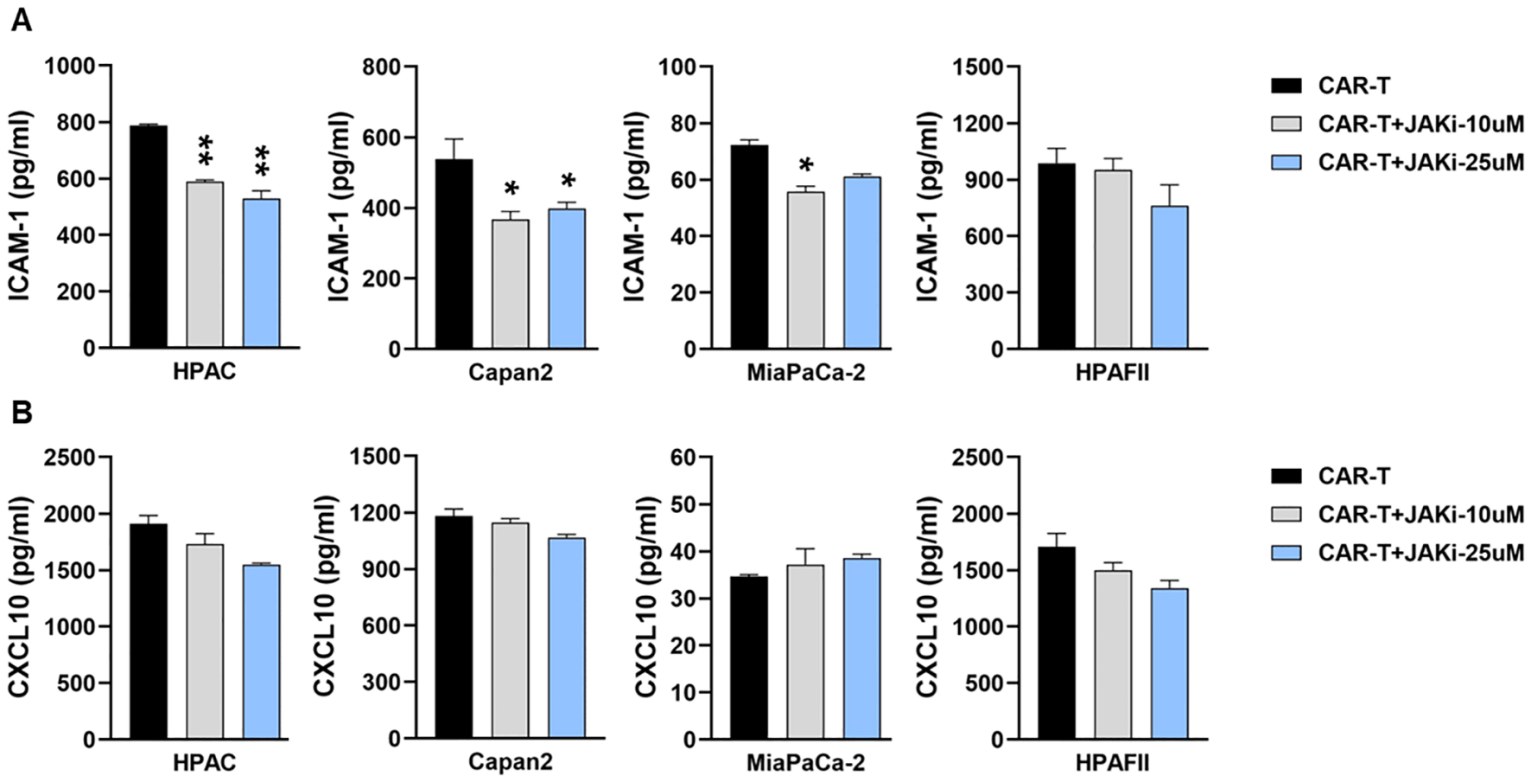
Suppressing IFN signaling in CAR T cells slightly decreases ICAM-1 level. PDA cell lines were plated overnight for attachment. On the same day, tMUC1-CAR T cells were treated with or without JAKi at 10μM and 25μM overnight. On the following day, JAKi-pretreated CAR T cells were washed with fresh media to remove JAKi before being added to PDA cells at E:T ratio of 5:1 for co-culture. After 24hr, co-culture supernatants were collected and assayed for cytokine levels of **(A)** ICAM-1 and **(B)** CXCL10 by ELISA. Data are presented as the mean ± SD of triplicate. The statistical comparison was conducted between JAKi-pretreated CAR T cell group and media-pretreated CAR T cell group in PDA. *p<0.05, **p<0.01 (unpaired t test with Welch’s correction).

Since T cells are also known to produce ICAM-1 and CXCL10 upon stimulation (42–44), we further examined their contribution to cytokine release. CAR T cells were pre-treated with or without JAKi (10 µM or 25 µM) overnight, thoroughly washed to remove residual JAKi, and then co-cultured with PDA cells for 24hr. As shown in **Figure 4**, pre-disrupting IFN signaling in CAR T cells only mildly reduced ICAM-1 levels in co-cultures with three PDA cell lines.

Therefore, data from **Figures 3** and **4** indicated that PDA cells were the primary source of ICAM-1 and CXCL10 in the co-cultures. The reduction in these cytokines following JAKi treatment highlighted the critical role of tumor-intrinsic IFN signaling in their production.

### IFN blockade regulates PDA intrinsic IFN signaling

**Figure 5:**
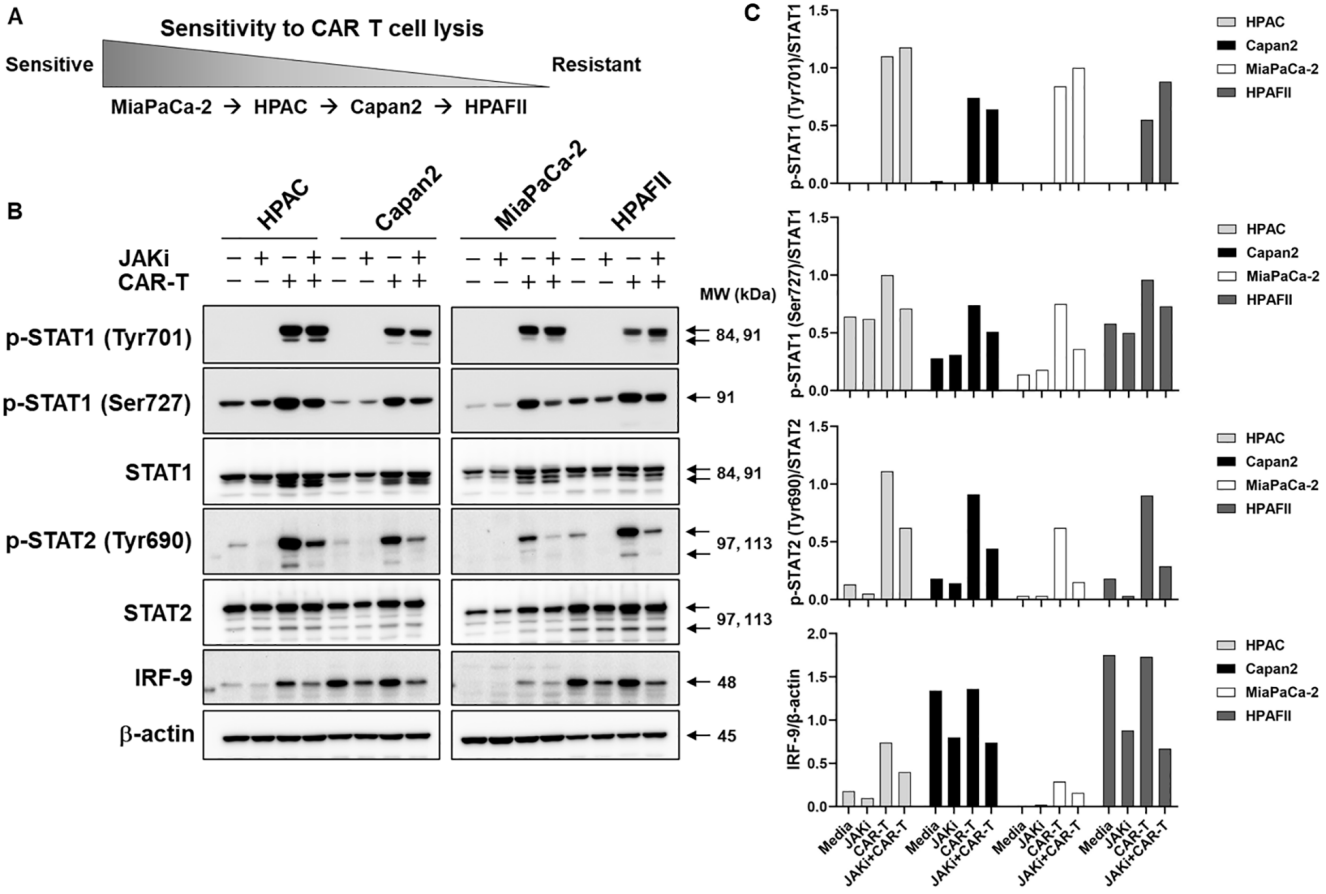
JAKi regulates IFN signaling in PDA. **(A)** Schematic representation of PDA cell line sensitivity to CAR T cell cytolysis. **(B)** PDA IFN signaling in response to CAR T cell challenge. PDA cell lines were plated overnight for attachment. On the next day, PDA cells were treated with JAKi at 25μM overnight. After JAKi treatment, the inhibitor was removed, and tMUC1-CAR T cells were added at E:T ratio of 5:1 in the absence of JAKi. The E:T ratio was calculated based on the initial number of PDA cells plated. After 3hr co-culture, CAR T cells were removed and whole cell lysates from attached live PDA cells were prepared. IFN signaling cascades were analyzed by Western Blot. Equal protein loading for each PDA cell lysate was confirmed by β-actin levels. MW, molecular weight. **(C)** The density of protein signal was quantified by ImageJ.

Data from **Figure 3** indicated a potential role for IFN signaling in PDA cytokine release. Building on this observation, we further investigated the presence of IFN signaling cascades within PDA cells and their regulation by IFN blockade in response to CAR T cell cytolysis. The four PDA cell lines were pretreated with or without JAKi (at 25μM) overnight. The following day, JAKi was removed, and PDA cells were co-cultured with CAR T cells for 3 hours. Adherent live PDA cells were then collected for Western blot analysis, and data was summarized in **Figure 5**. The sensitivity difference of PDA to CAR T lysis was restated in **Figure 5A** for signaling reference. Notably, MiaPaCa-2 cells displayed the weakest IFN signaling cascade expression. Treatment with JAKi effectively reduced baseline levels and/or CAR T cell-induced activation of type I and type II IFN signaling components, including phosphorylated STAT1 (p-STAT1 at Ser 727), phosphorylated STAT2 (p-STAT2 at Tyr690), and interferon regulatory factor 9 (IRF-9). Interestingly, IRF-9 and p-STAT2/STAT2 levels were endogenously higher in the resistant HPAFII and Capan2 cell lines, as well as in the less sensitive HPAC cells, compared to MiaPaCa-2 cells (**Figure 5B**). IRF-9, a transcription factor that partners with activated STAT1/2 to mediate type I IFN responses and downstream IFN-stimulated genes expression (45), together with p-STAT2, was dramatically suppressed by JAKi in PDA cells upon CAR T cell challenge.

Phosphorylated Jak1 (p-Jak1) and phosphorylated Jak2 (p-Jak2) were undetectable at this time point (**Supplementary Figure 3**). Interestingly, an increase in phosphorylated Tyk2 (p-Tyk2) was observed following JAKi treatment. This may result from JAKi’s inhibition of Jak1 phosphorylation, leading to the accumulation of unpaired p-Tyk2, which, without its typical pairing with p-Jak1, may hinder further cycling and dephosphorylation (**Supplementary Figure 3**).

### Blocking PDA-intrinsic IFN signaling enhances PDA sensitivity to CAR T cell killing

**Figure 6:**
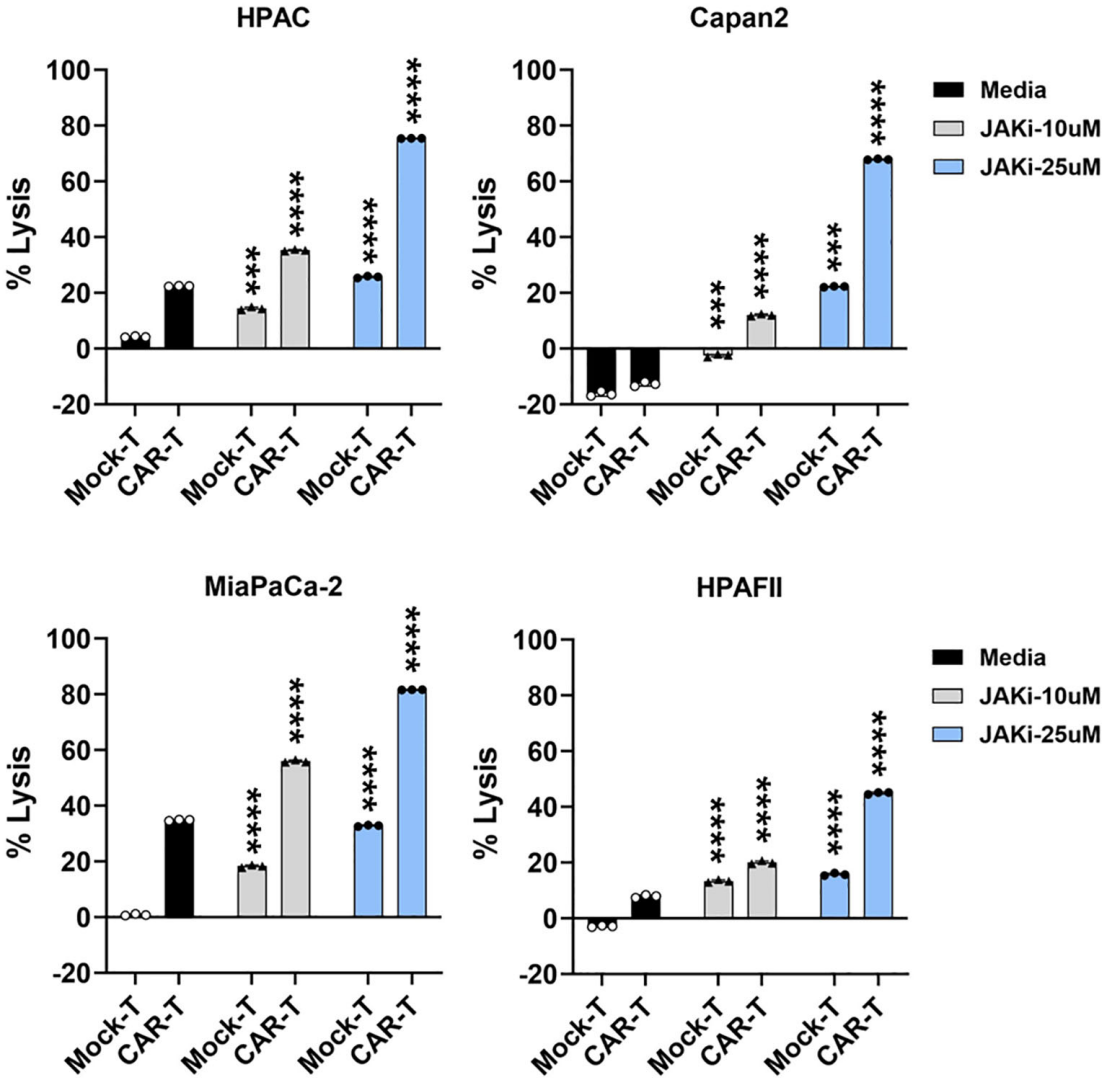
Blocking IFN pathway in PDA enhances its sensitivity to T cell-mediated killing. PDA cell lines were plated overnight for attachment. On the next day, PDA cells were treated with JAKi at 10μM and 25μM overnight. After JAKi treatment, the inhibitor was removed, and tMUC1-CAR T cells or Mock T cells were added in the absence of JAKi. All T cells were added at E:T ratio of 5:1, calculated based on the initial number of PDA cells plated. After 24hr, PDA cell lysis was assessed using MTT assay. Media control data were used for calculating the percentage of lysis with the formula: % lysis = [(OD of culture with media – OD of co-culture with CAR T or Mock T cells)/OD of culture with media] ×100. The statistical comparison was conducted between CAR T cell treatment in JAKi-pretreated PDA and CAR T cell treatment in PDA without JAKi pretreatment. The statistical comparison was also conducted between Mock T cell treatment in JAKi-pretreated PDA and Mock T cell treatment in PDA without JAKi pretreatment. ***p<0.001, ****p<0.0001 (Multiple unpaired t tests with Welch’s correction). Data are presented as the mean ± SD from triplicate.

Building on data from **Figures 3** and **5**, we further investigated the functional role of PDA-intrinsic type I/II IFN signaling in modulating PDA sensitivity to CAR T cell cytolysis. To assess this, PDA cells were pretreated with JAKi (10 µM or 25 µM) overnight to suppress IFN signaling, followed by co-culture with CAR T cells. As shown in **Figure 6**, JAKi pretreatment at 25 µM significantly enhanced the sensitivity of all four PDA cell lines to tMUC1-targeted CAR T cell killing. Interestingly, we also observed an increase in tumor lysis by non-targeting Mock T cells in JAKi-pretreated PDAs. JAKi alone (at both 10 µM and 25 µM) had minimal to no impact on PDA cell survival in this assay (data not shown). To evaluate the potential contribution of CAR T cell-intrinsic IFN signaling, CAR T cells were pretreated with JAKi overnight before being used to lyse PDA cells. However, this treatment did not significantly enhance PDA killing (**Supplementary Figure 4**). Together, these findings suggested that PDA-intrinsic IFN signaling might play an essential role in PDA resistance to CAR T cell-mediated cytolysis. Suppressing this signaling pathway effectively enhanced PDA susceptibility to both targeted and non-targeted T cell cytotoxicity.

### Knocking down IFN receptors in PDA enhances its sensitivity to CAR T cell cytolysis

**Figure 7:**
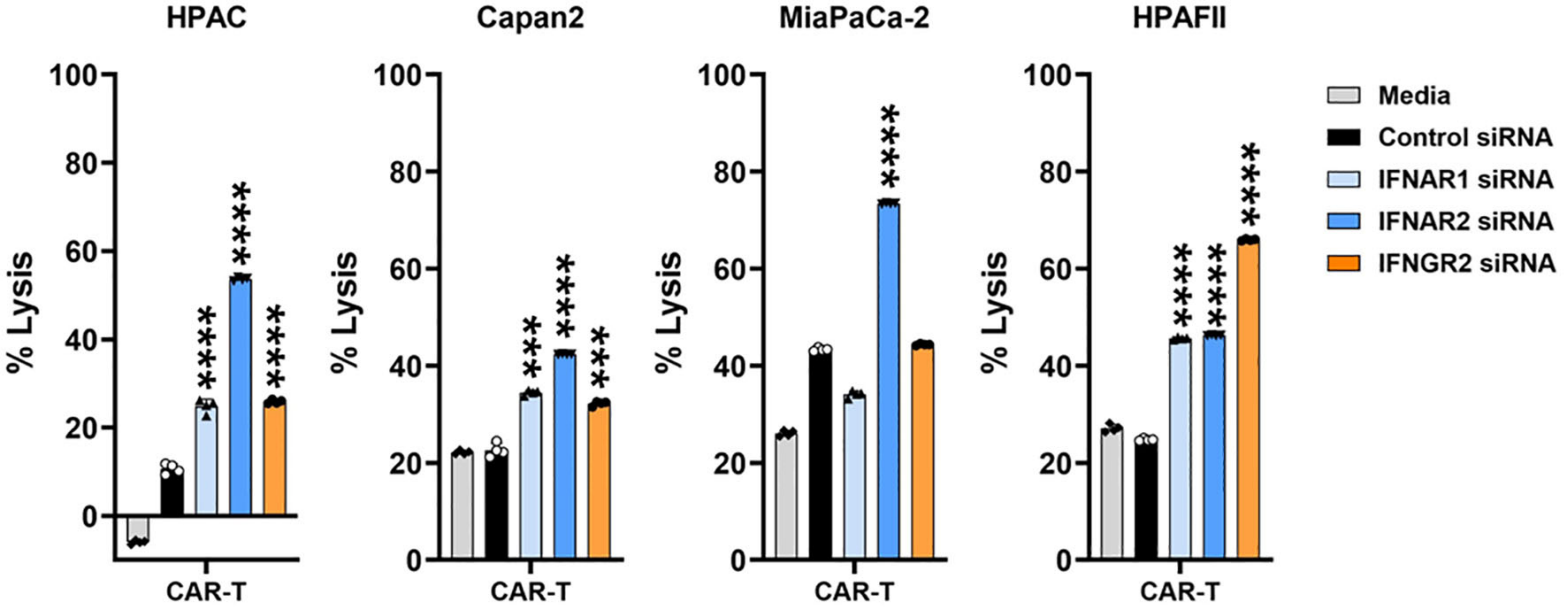
IFN receptor knockdown in PDA enhances its sensitivity to CAR T cell killing. PDA cell lines were plated overnight for attachment. On the following day, PDA cells were transfected with the indicated siRNA for 48hr. After siRNA treatment, the transfection medium was removed, and tMUC1-CAR T cells were added in the absence of siRNA. CAR T cells were added at E:T ratio of 10:1 for HPAC, Capan2, and HPAFII, and at E:T ratio of 2:1 for MiaPaCa-2 given its higher sensitivity to CAR T cell killing. The E:T ratio was calculated based on the initial number of PDA cells plated. After 24hr, PDA cell lysis was determined using MTT assay. Media control data were used to calculate the percentage of lysis. The calculation formula was: [(OD of culture with media – OD of co-culture with CAR T cells)/OD of culture with media] ×100. The statistical comparison was conducted between CAR T cell treatment in IFNR siRNA-pretreated PDA and CAR T cell treatment in control siRNA-pretreated PDA. ***p<0.001, ****p<0.0001 (unpaired t test with Welch’s correction). Data are presented as the mean ± SD from quadruplicate.

To further validate the findings from **Figure 6**, where pharmacological IFN blockade improved PDA sensitivity, we used siRNA to specifically knock down type I (IFNAR1 and IFNAR2) or type II (IFNGR2) IFN receptors in four PDA cell lines. The efficiency of siRNA knockdown was evaluated, as shown in **Supplementary Figure 5**. Following 48 hours of siRNA treatment, the siRNAs were removed from the PDA cultures, and CAR T cells were subsequently added for 24 hours. Consistent with the results observed using JAKi, siRNA-mediated downregulation of IFN receptors significantly enhanced PDA sensitivity to CAR T cell killing (**Figure 7**). These findings further supported the critical role of PDA-intrinsic IFN signaling in promoting resistance to CAR T cell-mediated cytotoxicity and highlighted IFN receptor targeting as a potential strategy to improve CAR T cell therapy.

### Tumor IFN signaling-induced immune checkpoints are involved in CAR T cell dysfunction

**Figure 8:**
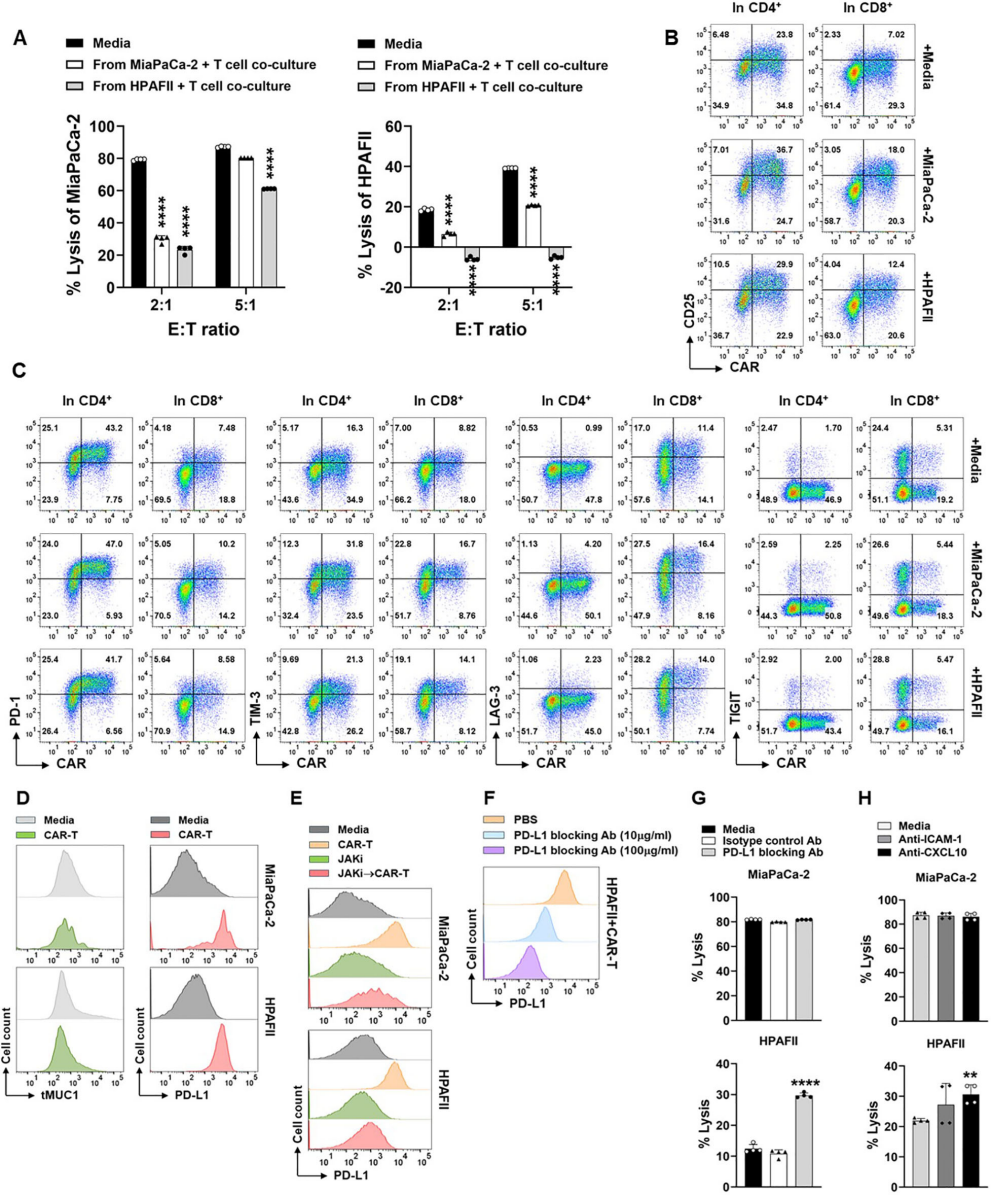
Engagement of CAR T cell with PDA induces function loss of CAR T cells and up-regulation of immune checkpoints. **(A)** Loss of CAR T cell cytotoxicity. CAR T cells or Mock T cells were co-cultured with MiaPaCa-2 or HPAFII cells at E:T ratio of 5:1 or cultured with media alone for overnight. Then the live CAR T cells and Mock T cells were isolated from the first round co-culture, and they were added to the fresh MiaPaCa-2 or HPAFII plates at E:T ratio of 2:1 or 5:1 for 24hr as the second round of co-culture. At the end of culture, tumor cell lysis against MiaPaCa-2 (left panel) or against HPAFII (right panel) was determined using MTT assay. The percentage of lysis was calculated using the formula: [(OD of co-culture with Mock T cells– OD of co-culture with CAR T cells)/OD of co-culture with Mock T cells] ×100. The mock T cells and CAR T cells are from the same pair for calculation. Data are presented as the mean ± SD from quadruplicate. The statistical difference was conducted for cytolysis of CAR T cells retrieved from co-culture with PDA cells when compared with cytolysis of CAR T cells retrieved from culture with media alone. ****p<0.0001 (Multiple unpaired t tests with Welch’s correction). **(B)** Increase of CD25 expression on CAR T cells after engagement with PDA. CAR T cells were co-cultured with MiaPaCa-2 or HPAFII cells at E:T ratio of 5:1 or cultured with media alone for overnight. Then CAR T cells were stained and analyzed for CD25 expression on CAR-positive and CAR-negative live cells after gating on CD4^+^ T cells and CD8^+^ T cells. **(C)** Expression of ICs on CAR T cells. Cell culture was performed same as in **(B)**. IC expression on CAR-positive and CAR-negative live cells were displayed after gating on CD4^+^ T cells and CD8^+^ T cells. **(D)** The retaining of tMUC1 and up-regulation of PD-L1 on PDA. After rinsing off suspension cells in co-culture from **(B)**, adherent MiaPaCa-2 or HPAFII cells were stained and analyzed for tMUC1 and PD-L1. PDA cells cultured in media alone were included as baseline control. **(E)** Suppressing tumor IFN signaling blocks CAR T cells-induced PD-L1 increase. MiaPaCa-2 or HPAFII cells were pre-treated with JAKi at 25μM overnight. Then JAKi was removed, and CAR T cells were added in the absence of JAKi at E:T ratio of 5:1, calculated based on the initial number of PDA cells plated. After 24hr treatment with CAR T cells, live adherent PDA cells were stained and analyzed for PD-L1 expression. **(F)** Effectiveness of PD-L1 blocking antibody. CAR T cells-treated HPAFII cells were pre-incubated with PD-L1 blocking antibody at the indicated doses, followed by PD-L1-PE staining. **(G)** Blocking PD-L1 partially reversed HPAFII resistance to CAR T cell lysis. MiaPaCa-2 or HPAFII cells were treated with CAR T cells or Mock T cells in the presence of PD-L1 blocking antibody or its isotype control for 24hr. PDA cell lysis was determined using MTT assay. Data are presented as the mean ± SD from quadruplicate. The statistical difference was conducted for cytolysis of CAR T cells with PD-L1 blocking antibody when compared with cytolysis of CAR T cells with media alone. ****p<0.0001 (unpaired t tests with Welch’s correction). **(H)** Involvement of ICAM-1 and CXCL10 in CAR T cell cytotoxicity. MiaPaCa-2 or HPAFII cells were treated with CAR T cells or Mock T cells in the presence of anti-ICAM-1 or anti-CXCL10 antibodies for 24hr. PDA cell lysis was determined using MTT assay. Data are presented as the mean ± SD from quadruplicate. The statistical difference was conducted for cytolysis of CAR T cells with neutralizing antibody when compared with cytolysis of CAR T cells with media alone. **p<0.01 (unpaired t tests with Welch’s correction).

We then used the sensitive MiaPaCa-2 cells and resistant HPAFII cells to further look into the downstream molecules post IFN signaling that could be responsible for PDA resistance. We first looked at CAR T cell changes in their function and phenotype. CAR T cells were retrieved from their 1^st^ round co-culture with PDA cells and then the live CAR T cells were used in the 2^nd^ round co-culture with PDA cells to evaluate their cytotoxicity function. Compared to the cytotoxicity of CAR T cells which were maintained in culture media, the lysis activity of CAR T cells recovered from their prior co-culture with MiaPaCa-2 or with HPAFII was significantly compromised, especially at lower E:T ratio of 2:1 (**Figure 8A**). Notably, the cytotoxicity of CAR T cell recovered from their co-culture with HPAFII was impaired the most.

To determine whether the suppression of cytotoxicity in CAR T cells after their co-culture with PDA was due to the lack of CAR T cell activation, CAR T cells from co-culture were analyzed for the level of CD25, a T cell activation marker. Compared to the CAR T cells cultured in media only, the CD25 expression was increased in CAR T cells after their culture with either MiaPaCa-2 or HPAFII cells, both in CAR-positive or CAR-negative populations in CD4 T cells or in CD8 T cells (**Figure 8B**).

We then analyzed the levels of immune checkpoint (IC) molecules on CAR T cells, including PD-1, TIM-3, LAG-3, and TIGIT. The presence of MiaPaCa-2 or HPAFII in culture did not significantly affect PD-1 and TIGIT expressions, but their presence increased the TIM-3 and LAG-3 expressions on CAR T cell surface when compared to CAR T cell cultured in media only (**Figure 8C**). From the same co-culture, we also analyzed the expression of tMUC1 and the IC molecule PD-L1 on PDA cells. There was no clear loss in tMUC1expression on cell surface of remaining live MiaPaCa-2 or HPAFII after their treatment with CAR T cells. However, there was dramatic up-regulation of PD-L1 expression on PDA cell surface after engagement with CAR T cells (**Figure 8D**). As we mentioned above in **Supplementary Figure 4**, pre-disruption of IFN signaling in CAR T cells did not significantly affect their cytotoxicity against PDA cells. Therefore, even though there was an increase in TIM-3 and LAG-3 on CAR T cells after their engagement with PDA cells, we preferred to manipulate the ICs from the PDA tumor side, in another word, on PD-L1 molecule.

To dissect the association of CAR T cell-triggered PD-L1 up-regulation with tumor intrinsic IFN signaling-induced PDA resistance, PD-L1 was analyzed in PDA cells which were pretreated with JAKi for overnight before they were challenged with CAR T cells. As shown in **Figure 8E**, CAR T cell-induced PD-L1 increase was greatly revoked in JAKi-pretreated MiaPaCa-2 and HPAFII cells. We then determined the functional contribution of PD-L1 increase to PDA resistance. The efficiency of PD-L1 blocking antibody was confirmed using a competition binding assay. PD-L1 blocking antibody at 10μg/ml or 100μg/ml were able to effectively block the binding sites of tumor cell surface PD-L1 (**Figure 8F**). In the cytotoxic function assay, PD-L1 blocking antibody partially rescued the CAR T cell killing in HPAFII cells (**Figure 8G**, bottom panel). PD-L1 blocking antibody did not further enhance CAR T lysis against MiaPaCa-2 since CAR T cells at E:T ratio of 5:1 already exerted a near maximal killing in MiaPaCa-2.

As shown in the above figures, the levels of released ICAM-1 and CXCL10 were much higher in resistant HPAFII cells. JAKi pretreatment strongly inhibited their production by HPAFII and other 3 PDA cell lines and at the same time enhanced those PDA cell response to CAR T cell lysis. We neutralized the ICAM-1 and CXCL10 during the PDA cell co-culture with CAR T cells. There was a trend of increased response of HPAFII to CAR T cell killing, with CXCL10 neutralization being significant (**Figure 8H**, bottom panel). Similar to PD-L1 blocking assay, neutralizing ICAM-1 or CXCL10 did not affect CAR T lysis against MiaPaCa-2 at the given condition.

### Poor prognosis is correlated with higher gene expression along tumor IFN signaling pathway in pancreatic cancer patients

**Figure 9:**
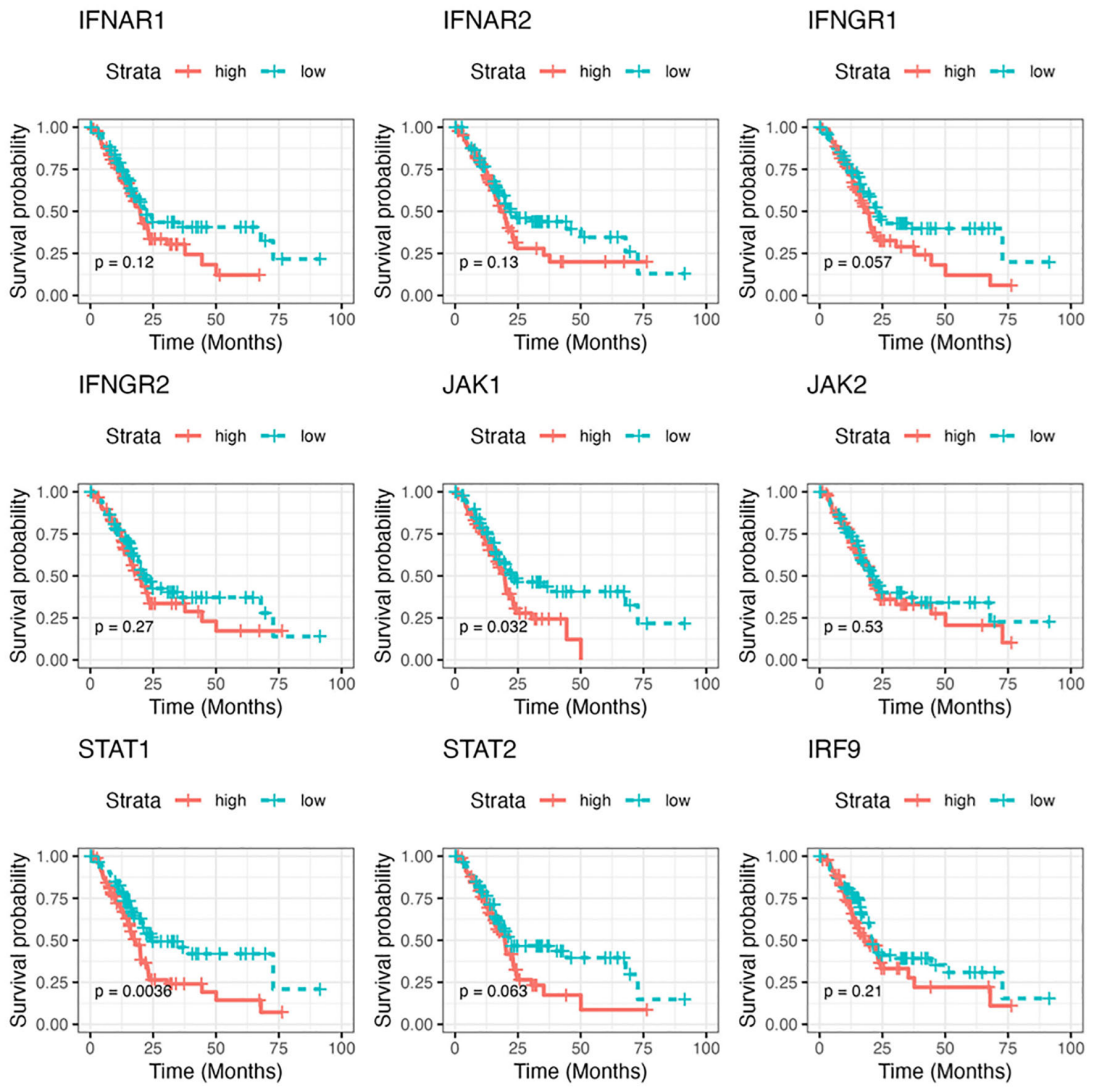
The correlation of gene expressions along the IFN pathway with patient prognosis in pancreatic cancer. The Kaplan-Meier survival curve of overall survival for pancreatic adenocarcinoma patients with low/high RNA levels of selected IFN signaling molecules. The analysis was performed based on RNA-seq gene count data in TCGA and by STAR (Spliced Transcripts Alignment to a Reference) analysis. The survival cures were plotted using survival package in R. P value less than 0.05 is considered significant.

Finally, to assess the clinical relevance of our findings, we chose several essential signaling cascade molecules along the IFN pathway and analyzed the levels of their gene expression in predicting the survival of patients with PDA. The RNA-seq gene count data in TCGA pancreatic adenocarcinoma project was utilized and gene expression was calculated and grouped as low expression group and high expression group. As shown in **Figure 9**, compared to the patients with lower gene expression, patients with higher expression of those selected genes were likely to have poorer survival probability, with JAK1 and STAT1 showing the statistically significant roles.

## Discussion

There are only very limited reports on the topics of IFN signaling involvement in immunotherapy resistance. To the best of our knowledge, our study will be the first to demonstrate the tumor IFN signaling as a novel PDA-intrinsic mechanism that contributes to PDA resistance to tumor MUC1-targeted CAR T cell therapy, and such mechanism may be universal and essential that most or all PDA tumors use in their resistance to tumor antigen-targeted T cell therapy beyond tMUC1-CAR T cells. And most relevantly, elevated tumor IFN signaling gene expression was correlated with poorer prognosis in patients with PDA, which might serve as a marker to predict the patients’ poorer response to therapeutic T cell treatments and lower overall survival rate.

The mechanisms of PDA resistance to CAR T cell therapies haven’t been well studied so far. A recent publication reported that tumor IFN-γ receptor1 signaling was required for CAR T cell killing efficacy in glioblastoma cells through regulating cell adhesion in CAR T-tumor cell interactions, in which the CAR T cell-induced but tumor-produced ICAM-1 played a critical role (23). Opposite from this report, our findings demonstrated that tumor-intrinsic IFN signaling played a pivotal role in mediating PDA resistance to tMUC1-CAR T cell cytolysis. By using HPAFII cells which had robust resistance to CAR T cell killing, we identified elevated ICAM-1, CXCL10, and CXCL11, which were associated with IFN signaling and primarily produced by PDA cells. Blocking ICAM-1 and CXCL10 during CAR T-PDA co-culture partially increased PDA lysis *in vitro*, with CXCL10 neutralization showing statistically significant.

Immune checkpoint blockade represents an exciting new modality of cancer immunotherapy. The failure of ICB has been ascribed to either the innate resistance to ICB treatment or ICB treatment-induced adaptive resistance, in which tumor type I and/or type II IFN signaling has been implicated (27–29). In our study, tumor-intrinsic type I/II IFN signaling was demonstrated to play an important role in PDA resistance to CAR T cell lysis. On the T cell side, CAR T cells remained activated as shown with CD25 expression, and they displayed increased expression of immune checkpoint TIM-3 and LAG-3 and compromised cytotoxicity after their engagement with PDA cells *in vitro*. On the tumor side, it is known that IFN, particularly IFN-γ, can induce expression of immune checkpoints such as PD-L1 and PD-L2 on both tumor and immune cells (46). In fact, our tMUC1-CAR T cell treatment triggered PD-L1 upregulation on PDAs which was largely dependent on the tumor intrinsic IFN signaling, and HPAFII responded to PD-L1 blocking antibody to show improved CAR T cell killing efficacy. We found that PDA intrinsic IFN signaling increased its own PD-L1 and CXCL10 to build its resistance to CAR T cell lysis. It is possible that additional downstream molecules along the IFN pathway are involved in PDA resistance. The detailed profiling of IFN signaling-regulated downstream gene/protein expression that are responsible for PDA resistance to CAR T cell treatment will be investigated separately.

The impact of tumor-intrinsic IFN signaling in PDA response to CAR T cell lysis has not been reported yet. Therefore, we would like to highlight our findings along the IFN signaling pathway. IFN blockade downregulated key IFN pathway molecules, including p-STAT1, p-STAT2, and IRF-9, in PDA cells upon CAR T cell challenge. These IFN signaling mediators were endogenously elevated in resistant PDA lines like HPAFII, Capan2, and HPAC compared to the more sensitive MiaPaCa-2. Specifically, the type I IFN signaling proteins p-STAT2 and IRF-9 were more affected by JAKi treatment, both at baseline and after CAR T cell challenge. STAT1, which is activated by both type I and type II IFN pathways, was also upregulated in response to CAR T cells at Tyr701 and Ser727 sites. However, the inhibition of JAKi on p-STAT1 (Tyr701) was less pronounced. This differential effect may reflect the distinct functions of the Tyr701 and Ser727 phosphorylation sites on STAT1, as previous research has indicated that phosphorylation at Tyr701 is necessary for DNA binding, while phosphorylation at Ser727 is required for effective gene transcription (45). Even though JAKi treatment reduced p-STAT1 (Ser727), the effect was not as significant as on p-STAT2. Thus, type I IFN signaling may play a leading role in PDA’s intrinsic resistance to targeted T cell therapy. Although p-STAT1 was less affected by JAKi, the potential contribution of type II IFN signaling could not be excluded. In fact, siRNA knockdown of type I (IFNAR1 and IFNAR2) and type II (IFNGR2) IFN receptors suggested that both IFN pathways contributed to PDA resistance.

The JAK-STAT3 signaling, which plays a key role in tumor cell survival, proliferation, migration, and tumorigenesis (47), is often implicated in various cancers (48, 49). In this study, JAKi was used to inhibit JAK1 and JAK2 activities of PDAs in order to block the subsequent IFN signaling. It’s possible that the potential JAK1 (or JAK2)-STAT3 signaling (50–52), different from IFN pathway, could also be affected. But we found that the effect of CAR T cells and JAKi on STAT3 activation was minimal (**Supplementary Figure 6).** This data further supported the primary role of tumor intrinsic IFN signaling in PDA resistance.

Both pharmacological IFN blockade and siRNA-mediated IFN receptor knockdown enhanced PDA sensitivity to CAR T cell killing. We recognize that the JAKi or siRNA knockdown were transient and likely insufficient for complete IFN blockade in tumors. To gain a deeper understanding of the role of tumor-intrinsic IFN signaling in PDA resistance, we are generating type I and type II IFN receptor knockout PDA cell lines using CRISPR/Cas9 gene editing. These sublines will enable us to separately and more precisely further assess the contribution of type I and type II IFN signaling to PDA resistance, both *in vitro* and in animal models in future studies.

Before we conclude this current study, we would like to point out a few limitations. One is the MTT assay we used to determine the cytotoxicity of CAR T cells. MTT assay measures the metabolic activity of live cells which is primarily mediated by mitochondrial enzymes. The MTT assay can reflect cell viability but has lower sensitivity in detecting viable cell numbers. The accuracy of MTT assay depends on cell seeding number with a limited linear range and cell metabolic activity (53). For the tumor-CAR T cell co-culture, MTT assay measures the whole metabolic activity in the co-culture, and thus is not able to accurately reflect the viable tumor cells. We recognize the residual presence of CAR T cells in the PDA-CAR T cell co-culture even though rigorous efforts had been made to remove T cells from co-culture before adding MTT. Therefore, other alternative and more sensitive assays are required. Another limitation is that each PDA cell line-specific CAR T cell resistance has not been fully disclosed, even though we demonstrated that tumor intrinsic IFN signaling and its downstream immune regulators might be an universal resistant mechanism utilized by PDA cells in general. Using of IFN receptor knockout PDA cell lines will help to further complete the mechanism along this pathway. The other limitation of our current study is that the *in vitro* findings have not been validated in *in vivo* tumor models.

Taken together, our data suggests that the cancer cell intrinsic IFN signaling drives immune evasion in PDA against tMUC1-specific T cell immunotherapy. Targeting tumor IFN signaling may present a novel therapeutic strategy to overcome PDA resistance to CAR T cell therapy.

## Data Availability

The original contributions presented in the study are included in the article/**Supplementary Material**, further inquiries can be directed to the corresponding authors.
