# Supplementary material for: Tumor-intrinsic interferon signaling drives pancreatic cancer resistance to tumor mucin1-targeted CAR T cell therapy
Source: Front Immunol. 2025 Aug 8;16:1618415. doi: 10.3389/fimmu.2025.1618415 (PMC12370727; doi:10.3389/fimmu.2025.1618415)

**Supplementary Materials**

**Figure legends**

**Supplementary Figure 1. tMUC1 CAR expression in transduced human T cells.** Human PBMCs from normal healthy donors were activated with anti-human CD3/CD28 antibodies for 3 days, followed by overnight CAR transduction with retrovirus supernatant. CAR expression was detected 7 days after transduction by flow cytometry analysis. Cells were gated for CD4 or CD8, and then analyzed for Myc-tag expression. Dead cells were excluded by 7-AAD staining. Mock-T, mock control T cells; CAR-T, tMUC1-specific CAR T cells.

**Supplementary Figure 2. Cytotoxicity of Ruxolitinib in PDAs.** The PDA cell lines were plated overnight in 96-well plate for cell attachment. On the following day, PDA cells were treated with JAK1/2 inhibitor Ruxolitinib at the indicated concentrations for 24hr. The PDA cell viability was determined using MTT assay. The percentage of lysis was calculated using the formula: [(OD of culture with media – OD of culture with Ruxolitinib)/OD of culture with media] ×100. Data are presented as the mean ± SD from quadruplicate.

**Supplementary Figure 3. Phosphorylation levels of Jak1/Jak2/Tyk2.** The same cell lysates from Figure 5 were also analyzed for levels of Jak1, Jak2, Tyk2 and their phosphorylation by Western Blot. The p-Jak1 and p-Jak2 were not detectable at this experiment point. Please note that the same β-actin data from Figure 5 is included here for equal loading reference. The density of protein signal was quantified by ImageJ.

**Supplementary Figure 4. Blocking IFN signaling in CAR T cells does not affect its cytotoxicity against PDAs.** PDA cell lines were plated overnight for attachment. On the same day, tMUC1-CAR T cells were treated with or without JAKi at 10μM and 25μM overnight. On the following day, JAKi-pretreated CAR T cells were washed with fresh media to remove JAKi before being added to PDA cells at E:T ratio of 5:1 for co-culture. After 24hr, PDA lysis was determined using MTT assay. Media control data were used for calculating the percentage of lysis with the formula: % lysis = [(OD of culture with media – OD of co-culture with CAR T cells)/OD of culture with media] ×100.

**Supplementary Figure 5. Efficiency of IFN receptor knockdown by siRNA.** The PDA cell lines were transfected with the indicated siRNA for 48hr and 72hr. The cell lysates were analyzed for the levels of IFNAR1 (72hr after siRNA) and IFNAR2 (48hr after siRNA) by Western Blot. The IFNR levels were very low for detection in Capan2 and HPAFII, so their data are not shown. Since the level of IFNGR2 was low and not clear in all 4 PDAs for detection by WB, the confirmation of IFNGR2 knockdown is not shown here. The density of protein signal was quantified by ImageJ.

**Supplementary Figure 6. JAKi does not significantly affect STAT3 signaling.** The same cell lysates from Figure 5 were also analyzed for levels of STAT3 and its phosphorylation by Western Blot. The density of protein signal was quantified by ImageJ.

**Supplementary Figure 1**


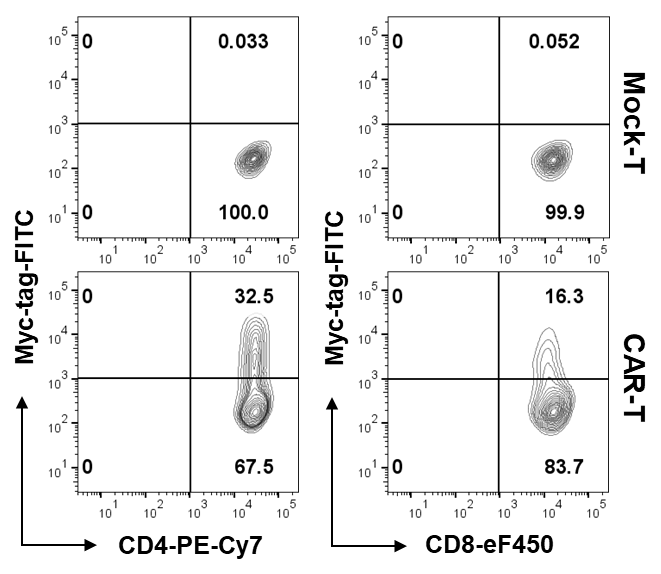


**Supplementary Figure 2**


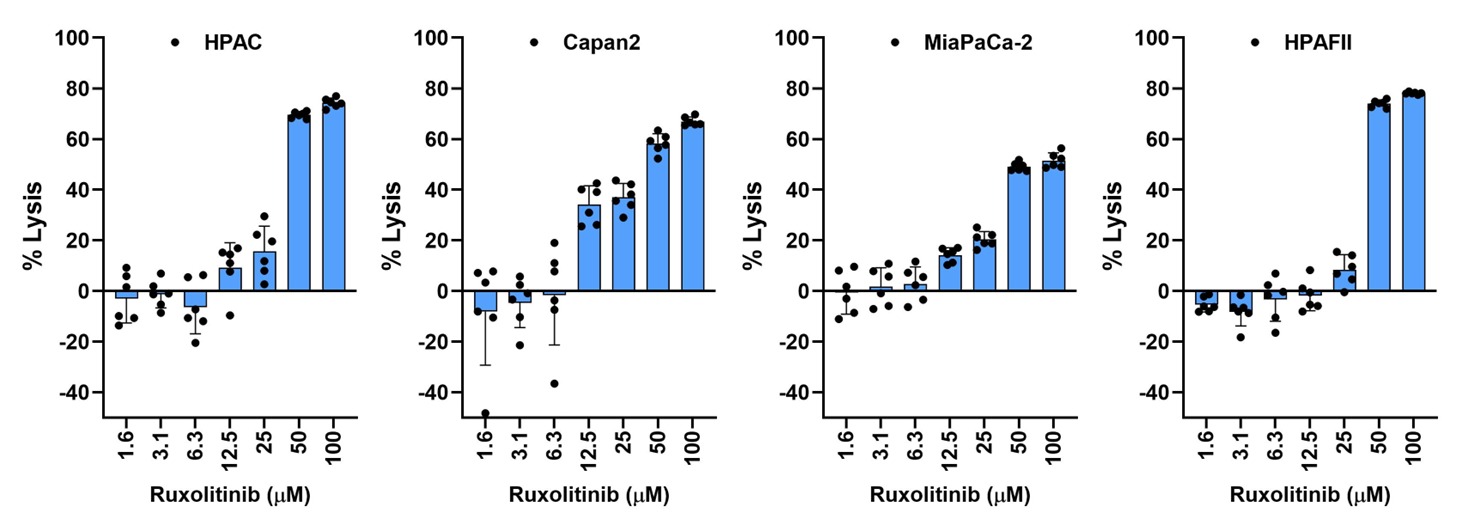


**Supplementary Figure 3**


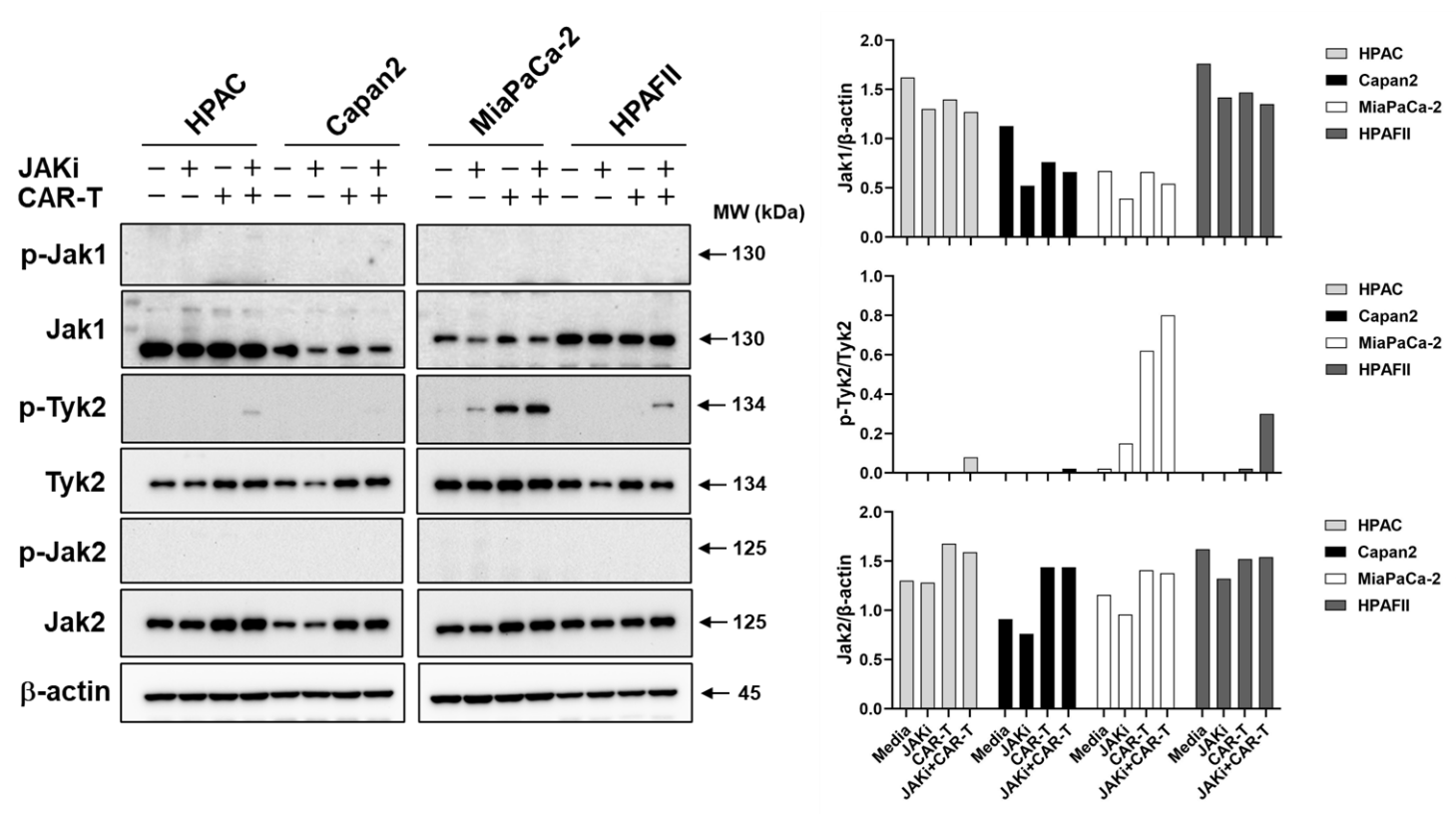


**Supplementary Figure 4**

**
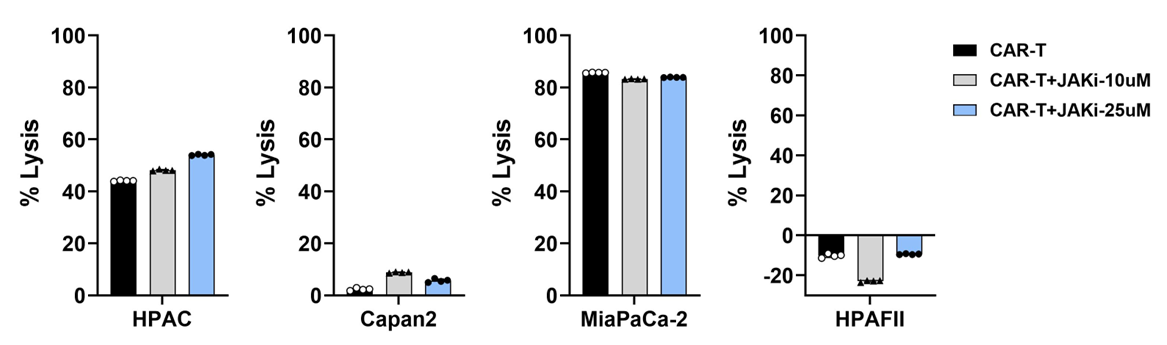
**

**Supplementary Figure 5**


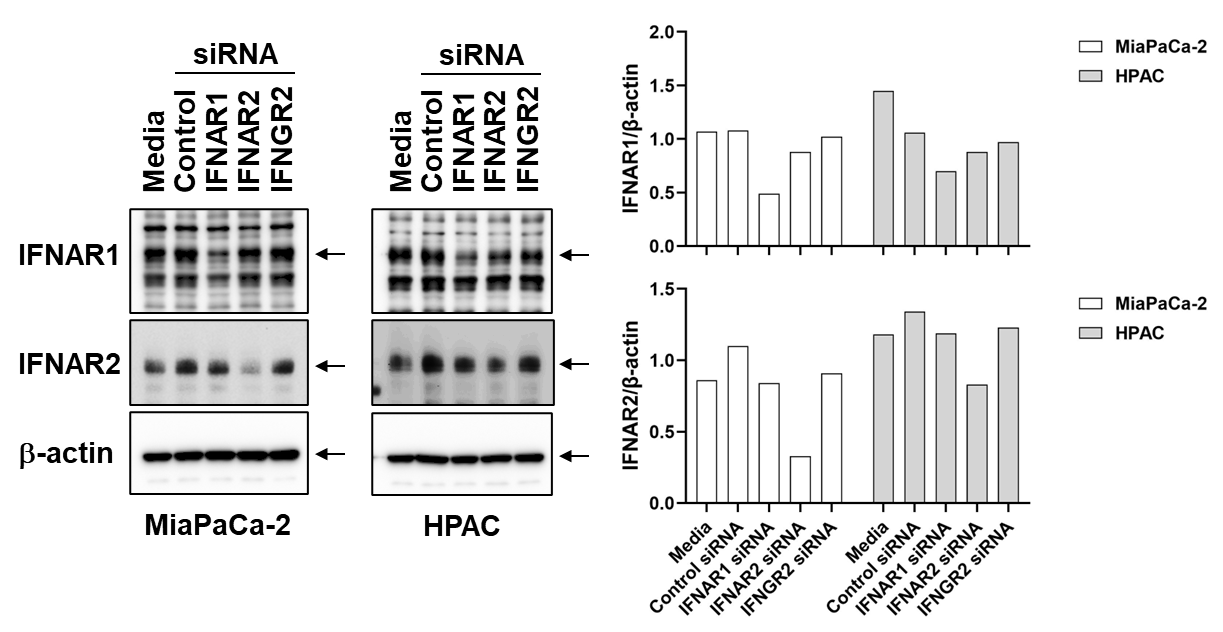


**Supplementary Figure 6**


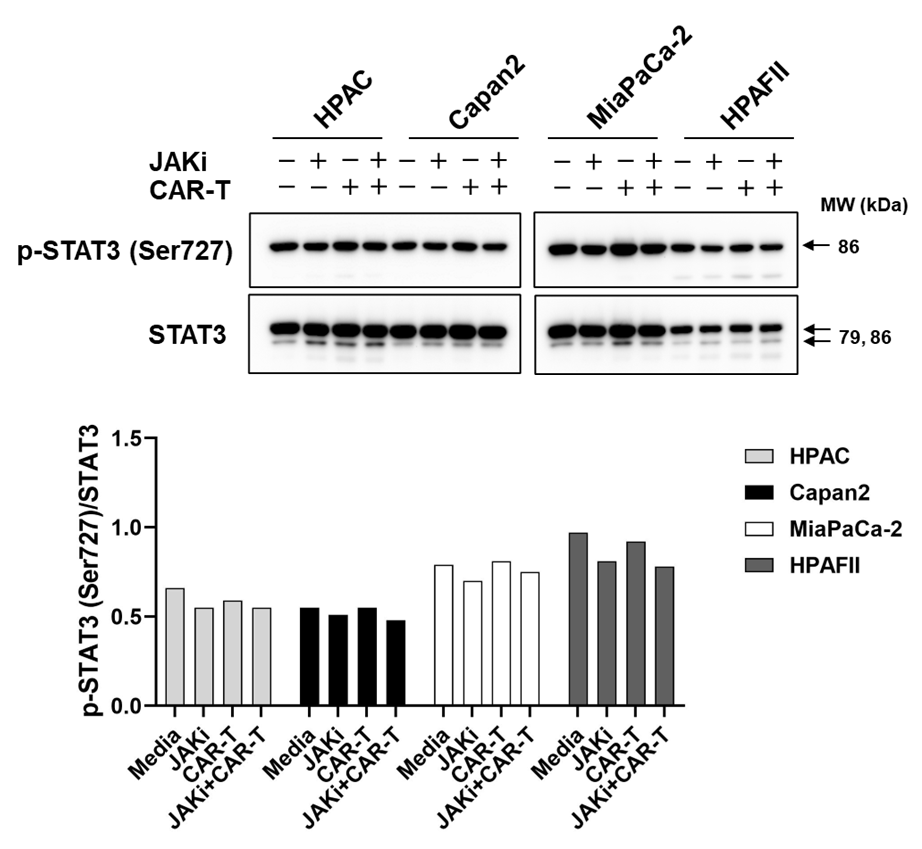

Supplement: Supplementary file 1 [file DataSheet1.docx]
